# Merck's Actions Surrounding Vioxx

**DOI:** 10.1371/journal.pmed.0030286

**Published:** 2006-06-27

**Authors:** Michael Heinley

**Affiliations:** **1**Merck & Company, Whitehouse Station, New JerseyUnited States of America

Merck has always been committed to the highest standards of scientific integrity, patient safety, and ethics. In his part of the *PLoS Medicine* Debate entitled “What Are the Public Health Effects of Direct-to-Consumer Drug Advertising,” Richard Kravitz incorrectly characterizes Merck's actions surrounding Vioxx [ 1].

Any suggestion that Merck acted improperly in the development and marketing of Vioxx is simply false. Vioxx was a widely used medicine because it served as an effective therapy for patients for whom—in many instances—no other alternative medicine worked. Merck's marketing efforts for Vioxx provided balanced and accurate information about both the product's considerable benefits for patients living with chronic pain as well as its potential risks.

Merck remains committed to producing innovative, safe, and therapeutic medicines for patients and to upholding the highest standards of scientific integrity. Our reputation in the industry, with patients and our community, is that of a company that puts patients first. We intend to keep it that way.

To learn more about Merck's actions, please visit the Vioxx Information Center on our Web site at http://www.merck.com/newsroom.
